# Loss of variation of state detected in soybean metabolic and human myelomonocytic leukaemia cell transcriptional networks under external stimuli

**DOI:** 10.1038/srep35946

**Published:** 2016-10-24

**Authors:** Katsumi Sakata, Toshiyuki Saito, Hajime Ohyanagi, Jun Okumura, Kentaro Ishige, Harukazu Suzuki, Takuji Nakamura, Setsuko Komatsu

**Affiliations:** 1 Maebashi Institute of Technology, Maebashi 371-0816, Japan; 2 National Institute of Radiological Sciences, Chiba 263-8555, Japan; 3 King Abdullah University of Science and Technology, Thuwal 23955-6900, Kingdom of Saudi Arabia; 4 RIKEN Centre for Life Science Technologies, Yokohama 230-0045, Japan; 5 National Agriculture and Food Research Organisation (NARO) Hokkaido Agricultural Research Centre, Sapporo 062-8555, Japan; 6 NARO Institute of Crop Science, Tsukuba 305-8518, Japan

## Abstract

Soybean (*Glycine max*) is sensitive to flooding stress, and flood damage at the seedling stage is a barrier to growth. We constructed two mathematical models of the soybean metabolic network, a control model and a flooded model, from metabolic profiles in soybean plants. We simulated the metabolic profiles with perturbations before and after the flooding stimulus using the two models. We measured the variation of state that the system could maintain from a state–space description of the simulated profiles. The results showed a loss of variation of state during the flooding response in the soybean plants. Loss of variation of state was also observed in a human myelomonocytic leukaemia cell transcriptional network in response to a phorbol-ester stimulus. Thus, we detected a loss of variation of state under external stimuli in two biological systems, regardless of the regulation and stimulus types. Our results suggest that a loss of robustness may occur concurrently with the loss of variation of state in biological systems. We describe the possible applications of the quantity of variation of state in plant genetic engineering and cell biology. Finally, we present a hypothetical “external stimulus-induced information loss” model of biological systems.

For more than 50 years, biological systems have been studied as general systems^1^,^2^,^3^,^4^,^5^,^6^,^7^,^8^. In such studies, components of the biological systems were quantified to generate a mathematical model. The basis of quantification included thermodynamics entropy^1^, hierarchy structure^2^, and automaton^3^ in classical studies published before the 1960s, and pathway structure^4^, reaction system^5^, regression model based on meteorological variables^6^, noisy information processing^7^, and information flow in a signal transduction system^8^ in studies published more recently. A criticism of these studies is that biological information is too complex to quantify meaningfully; for example, some intrinsic information underlies developmental processes^9^, and the phenotype is specified by the DNA genotype^10^.

Plant abiotic stress responses have been studied using ‘omics’ approaches^11^,^12^. Soybean (*Glycine max*) is sensitive to flooding stress, and flooding damage at the early seedling stage is a critical factor that affects production^13^. (Growth suppression in soybean seedlings after flooding is documented in the Supplementary Note). Multiple factors associated with gene transcription and protein abundance have been studied as candidate genetic targets related to the flooding response mechanism, based on ‘omics’ studies^12^. Cell differentiation processes have been studied in relation to recurrent gene expression patterns in transcriptional networks, which have been interpreted as cell types^14^,^15^. Human THP-1 myelomonocytic leukaemia cells cease proliferation, become adherent, and differentiate into immature monocyte- and macrophage-like phenotypes when stimulated with the tumour promoter, phorbol myristate acetate (PMA)^16^,^17^.

In the Gene Ontology framework, the term ‘response to’ is defined as a process that results in a change in the state or activity of a cell or organism as the result of a stimulus^18^. In this study, we introduce a quantity measure of biological systems that defines their variation of state, and quantify the states that the system can maintain in response to stimuli. We show that the variation of state is lost under external stimuli in two biological systems, regardless of the type of regulation (metabolism or transcription) and stimulus (flooding or PMA). We then applied the quantity of variation of state to plant genetic engineering and cell biology. Finally, we present a hypothetical “external stimulus-induced information loss” model of biological systems. Our simulation experiments were based on metabolomic and transcriptomic experimental data, and the results were partially validated by proteome data. Our hypothetical model of biological systems was generated based on the results of simulation experiments.

## Results

### Quantification of variation of state and robustness in biological systems

We characterised the biological systems using two quantities: variation of state and robustness. We calculated the variation of state using the Shannon entropy (*H*) from the system-state of biological systems as follows^19^:


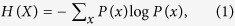


where *X* is a discrete system-state with possible values *x*. The Shannon entropy (also called information entropy) quantifies the average amount of information in system-states that the biological system can maintain. For example, for a system in which *P*(*x*_1_) = 1 for a system-state *x*_1_ and *P*(*x*_*i*_) = 0 for the other system-states *x*_*i*_ (*i* = 2, …, *n*), the value of *H* becomes 0 in equation (1). Conversely, if all the system states *x*_*i*_ (*i* = 1, …, *n*) appear in an equal probability *P*(*x*_*i*_) = 1/*n*, the value of *H* becomes the maximum value −log(1/*n*) (mathematical proof is provided in the Supplementary Note). These examples show that *H* represents the variation of states that the system can maintain. In environmental studies^20^,^21^, the second quantity, robustness, is defined as the degree to which a system is not influenced by external stimuli. Based on this definition, we calculated robustness (*R*) from the arithmetic mean of the states of modules in a biological system as follows:


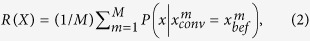


where *X* is a discrete system-state with possible values *x*, *m* is the number of modules in the biological system (1 ≤ *m* ≤ *M*), 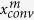 is the state of the *m*^*th*^ module at the final time of observation, and 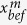 is the state of the *m*^*th*^ module without perturbation at the initial time of observation. The *R* value lies between 0 and 1.

Briefly, our aims were to: (i) simulate the behaviour of a biological system before and after external stimulation; (ii) describe the state trajectories of the biological system based on Boolean representation^22^ of the system-state variable; (iii) calculate variation of state and robustness from the state trajectories; and (iv) compare variation of state and robustness in simulated cases before and after stimulation.

### Simulation experiments in soybean metabolic network

**Figure 1:**
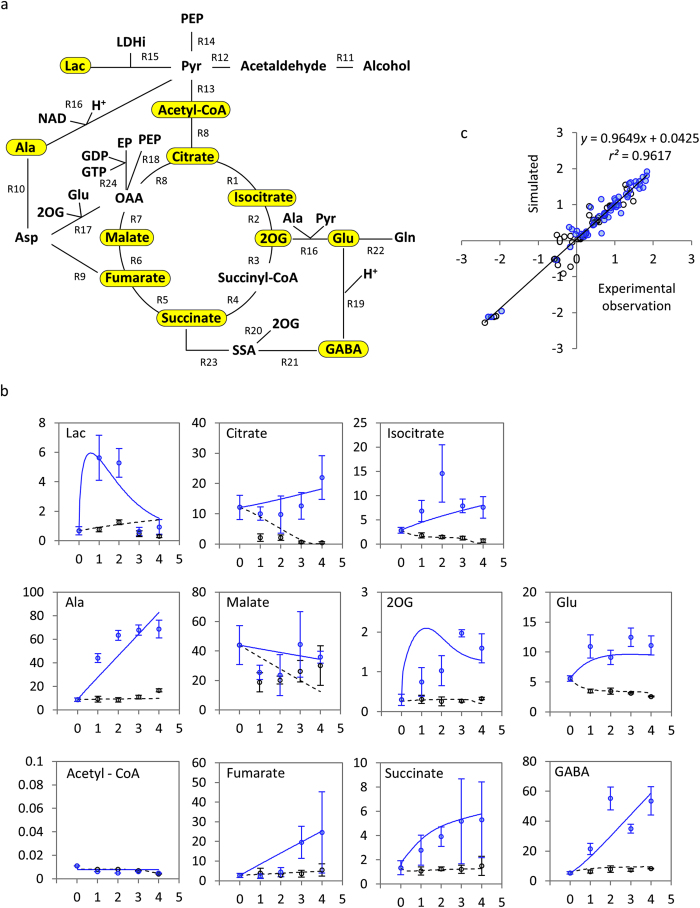
Soybean metabolic system model. (**a**) Schematic of soybean metabolic system. ‘Rx’ indicates reaction number in the metabolic data (reaction details are documented in Supplementary Table S1). Eleven fitting-target metabolites are shown in yellow. (**b**) Temporal profiles of experimentally measured and simulated amounts of metabolites without perturbations. Black and blue circles indicate experimentally measured amounts of metabolites in the control and flooding treatment, respectively. Error bars show standard error. Black dashed and blue solid lines indicate simulated amount of metabolites in the control and flooded models, respectively. Horizontal and vertical axes show days after the start of flooding treatment and number of moles (μmol/g dry weight), respectively. (**c**) Log–log plot of experimental versus simulated data for 11 fitting-target metabolites. Black and blue circles show data for control and flooded models, respectively. Abbreviations used: 2OG, 2-oxoglutarate; Ala, alanine; Asp, aspartic acid; EP, enolpyruvate; GABA, γ-aminobutyric acid; GDP, guanosine 5′-diphosphate; Gln, glutamine; Glu, glutamate; GTP, guanosine 5′-triphosphate; Lac, lactate; LDHi, lactate dehydrogenase inhibitor; NAD, nicotinamide adenine dinucleotide; OAA, oxaloacetate; PEP, phosphoenol pyruvate; Pyr, pyruvate; SSA, succinic semialdehyde.

The soybean metabolic data (see Methods) were mapped onto the glycolytic pathway, fermentation pathway, the tricarboxylic acid cycle, and the γ-aminobutyrate (GABA) shunt^23^. Previous studies have shown that GABA is produced rapidly and in large quantities in response to biotic and abiotic stresses^13^, and is metabolised mainly via a pathway that includes 2-oxoglutarate (2OG), glutamate (Glu), GABA, and succinate (Fig. 1), known as the GABA shunt^24^,^25^. The accumulation of GABA in stressed plant tissues has been interpreted as a link between the perception of an environmental stress and the physiological response^26^. Our previous experimental data showed that 2OG, Glu, GABA, and succinate accumulated during flooding^23^. Here, we investigated the accumulation of 2OG, Glu, GABA, and succinate by simulation experiments.

First, we constructed a differential equation model of the soybean metabolic system (Fig. 1a) that included and overlapped the glycolytic pathway, the fermentation pathway, the tricarboxylic acid cycle, and the GABA shunt, based on the metabolic data^23^. The assumed functions and kinetic parameters after an adjustment are documented in Supplementary Tables S1–S3. In the model, we set the Michaelis constants (*K*_*m*_ values documented in Supplementary Table S1) to the values listed in the enzyme database BRENDA^27^. We selected the *K*_*m*_ value for each reaction from BRENDA as follows: we found one or more *K*_*m*_ values for the corresponding enzyme; if there were multiple candidate *K*_*m*_ values, we selected the value for the enzyme in the species most closely related to soybean and in conditions most closely related to flooding stress. We set the maximum velocity (*V*_*max*_ values documented in Supplementary Table S1) as follows: we generated two models, a control model and a flooded model, using the metabolic pathway simulation program Winbest-kit^28^. During modelling, we fitted the simulated amount of 11 fitting-target metabolites (yellow coloured circles in Fig. 1a) to the metabolomic experimental data in the control and flooding conditions by adjusting the *V*_*max*_ values in each model. After these adjustments, the log-transformed simulated data and experimental data of the fitting-target metabolites were plotted against each other (Fig. 1c). A coefficient of determination (*r*^2^) value of 0.96 indicated that the model predictions were in close agreement with the observed data. The complete control and flooded models in Winbest-kit program are available in Supplementary Data 1 and Data 2.

**Figure 2:**
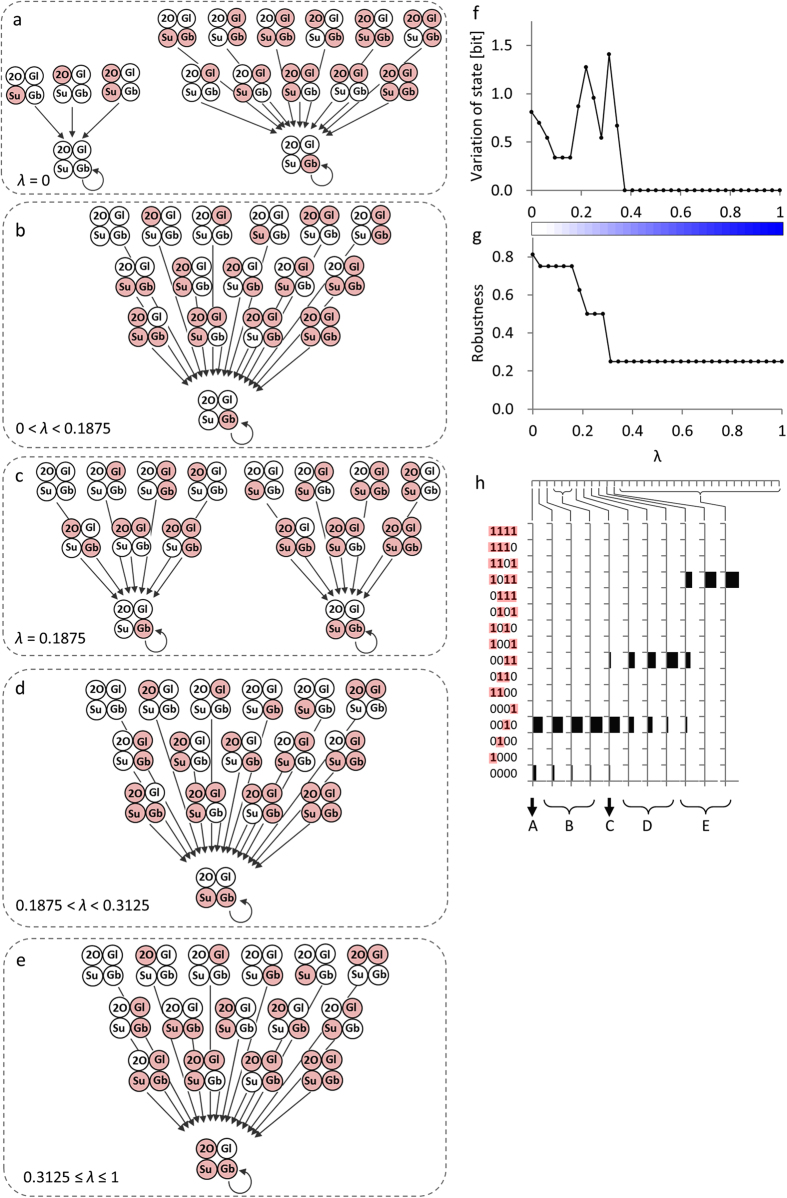
State representation of GABA shunt under flooding stimulus. (**a**–**e**) Initial and final states in state trajectories in simulation results. In each pattern, upper left, upper right, lower right, and lower left show accumulation state of 2OG, Glu, GABA, and succinate, respectively (red coloured circle: accumulated, open circle: not accumulated). *λ* indicates degree to which the model approximates flooded model. State positioned at bottom of each panel has an arrow back to itself, meaning that the initial and final states are the same in the corresponding state trajectory. Abbreviations used: 2O, 2OG; Gl, Glu; Gb, GABA; Su, succinate. (**f**) Variation of state measured as bit versus λ. (**g**) Robustness versus λ, the degree to which the model approximates the flooded model. Blue density in the colour bar between panels f and g represents degree to which the model approximates the flooded model. (**h**) State distribution during simulation period. Binary numbers in left column indicate the state of 2OG, Glu, GABA, and succinate accumulation, from left to right (1 (red coloured): accumulated, 0: not accumulated). Simulation period was 4 days and sampling interval was 1 day.

Second, we conducted simulation experiments and investigated the accumulation of the metabolites in the GABA shunt (2OG, Glu, GABA, and succinate) using our differential equation model. The time-courses in the simulations reflect the case in which 2-day-old soybean plants are flooded for 4 days in the flooded model and not flooded for 4 days in the control model. These conditions were same as those used to collect experimental data from soybean (see Methods). We described the simulation results as the state of the accumulation of the four metabolites based on Boolean representation. The Boolean value ‘true’ represented ‘accumulated’ (more than double the initial amount of the metabolite documented in Supplementary Table S2) and ‘false’ represented ‘not accumulated’ (no increase or an increase to less than double the initial amount documented in Supplementary Table S2). We defined the state of soybean under flooding stimulus using 2^4^ = 16-tuple the Boolean value, accumulation or not, of the four metabolites. In the simulation experiments, we set the initial values of the four metabolites, 2OG, Glu, GABA, and/or succinate to double their initial values (documented in Supplementary Table S2) as perturbations in the differential equation model (thus, we have 2^4^ = 16 cases of perturbation), and simulated the accumulation of the metabolites over 4 days (state trajectories documented in Supplementary Data 3). We also altered the simulation model by changing the maximum velocity, *V*_*max*_, of every reaction in the model (documented in Supplementary Table S1) linearly between the value of *V*_*max*_ in the control model (*λ* = 0) and the flooded model (*λ* = 1): *V*_*max*_ (λ) = *V*_*max*_ (λ = 0) + λ ∙ {*V*_*max*_ (λ = 1) − *V*_*max*_ (λ = 0)}. Here, *λ* indicates the degree to which the model approximates the flooded model, in other words, the degree to which the soybean response model approximates the model in flooded conditions. Using each altered model (*λ* = 0, 1/32, 2/32, …, 31/32, 1), we calculated the amount of metabolites and investigated the effects of changing *V*_*max*_ on the model’s behaviour. The accumulation state of 2OG, Glu, GABA, and succinate in the GABA shunt was indicated by Boolean representation, and the initial and final accumulation state of the four metabolites was plotted (Fig. 2a–e, the method is explained in Supplementary Methods). As indicated by *λ*, changing the model transformed the initial and final accumulation state patterns between the control and flooded model. The accumulation of metabolites was enhanced such that the number of accumulated metabolites in the final state increased according to changes in the model that approximated the flooded model (Fig. 2a–e). We calculated the variation of state from the simulated state trajectories (see Supplementary Methods). The median of variation of state was 0.68 bit in the region *λ* < 0.375, but lower in the region *λ* ≥ 0.375 (Fig. 2f). A Wilcoxon rank sum test^29^ confirmed a significant difference in the median of the variation of state between the regions *λ* < 0.375 and *λ* ≥ 0.375 (*p* < 10^−6^). The variation of state was calculated as 0.81 bit before and 0 bit after the flooding stimulus (Fig. 2f). The data showed a complete loss (20% of the theoretical maximum) of the variation of state. The robustness gradually decreased according to the values of *λ*, which is the degree to which the model approximates the flooded model, and the value was unchanged at 0.25 in the region *λ* ≥ 0.3125 (Fig. 2g). These results suggested that a loss of robustness occurred concurrently with the loss of variation of state in the model approximating the flooded model (Fig. 2f,g).

### Simulation experiments in human THP-1 myelomonocytic leukaemia cell transcriptional network

**Figure 3:**
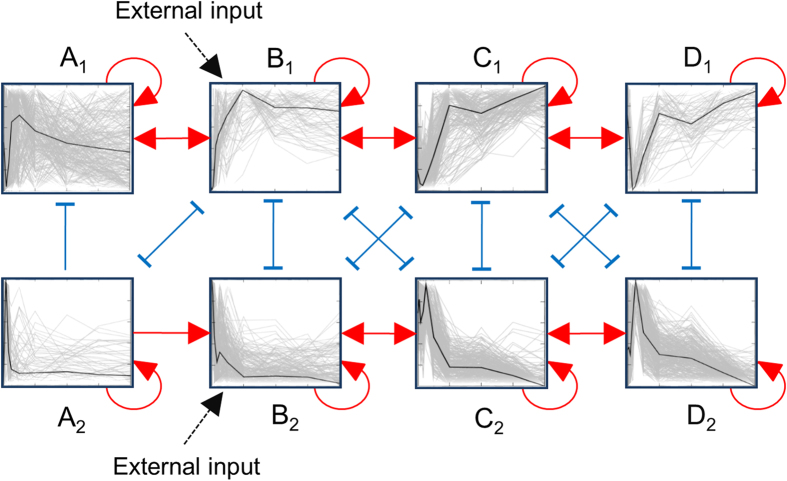
Transcriptional network model in human myelomonocytic leukaemia cells^15^. Eight transcription factor (TF) modules are connected by statistically significant inter-modular interactions. Red arrows and blue T-bars indicate promoting and inhibitory inter-modular interactions, respectively. Normalised temporal expression profiles of TFs are indicated in each module. Horizontal axis in each graph indicates time after starting external phorbol myristate acetate (PMA) stimulation, 0–96 h, and vertical axis indicates normalised expression values. Grey lines indicate normalised temporal profiles of TFs, and black lines indicate representative profile for each module defined as a series of medians. Numbers of TFs assigned to modules A_1_, A_2_, B_1_, B_2_, C_1_, C_2_, D_1_, and D_2_ were 310, 67, 76, 247, 196, 276, 71, and 376, respectively. In the modelling, first, TFs were grouped into clusters based on the goodness-of-fit of the interaction to the co-expression model^31^, i.e., two TFs that similarly interacted with third-party TFs were grouped together. As a result, four TF clusters were identified. Second, we conducted a second clustering using a k-means clustering with k = 2, based on temporal expression profiles. As a result, four TF clusters composed of two types of modules were identified; one roughly showing an upward trend (A_1_, B_1_, C_1_ and D_1_), and the other showing a downward trend (A_2_, B_2_, C_2_ and D_2_). Third, the identified modules were associated with inter-modular interactions as follows: the 80,540 interactions were identified as promoting or inhibitory based on a predicted value of a coefficient in the co-expression model^31^, and anchored to a combination of the two modules that included source TFs and sink TFs. A two-sample test^35^ for equality of proportions of the interactions identified 19 promoting and 17 inhibitory inter-modular interactions as statistically significant. Connection of eight TF modules with statistically significant inter-modular interactions revealed system-wide structure resembling two channels bridged by interfaces^15^.

In the human THP-1 myelomonocytic leukaemia cell transcriptional network, a system-wide, ladder-like transcription factor cluster structure was uncovered through co-expression-based analyses^15^. In the structure, 1619 transcription factors (TFs) were selected from the 2247 TFs (see Methods) as being relevant to the transcriptional network of human myelomonocytic leukaemia cells based on a covariance-based index^30^. In total, 80,540 interactions connecting the 1619 filtered TFs were identified based on the co-expression model^31^. In the structure, eight modules of TFs were identified, and the eight modules were associated with 19 promoting and 17 inhibitory statistically significant (*p* < 0.005) inter-modular interactions as the system-wide, ladder-like TF cluster structure (Fig. 3). The similarity of the temporal profiles was evaluated between a representative profile of each module and a unit step function that modelled the external input by PMA, which was applied at the beginning of the simulations and supplied continuously over the entire experimental period (see Methods). The B_1_ and B_2_ modules in the upper and lower channels in the modular structure of the system model (Fig. 3) showed the highest similarities among the modules. The further away a module was from B_1_ and B_2_, the lower the similarities of the module became. The positioning of the modules (Fig. 3) was suggested to be reasonable based on the idea that the temporal profile of an external input will be deformed as the external input is processed in a channel.

We conducted simulation experiments based on these modules to validate the state trajectories of the human THP-1 cells transcriptional network. To simulate the PMA treatment, we supplied step functions, 0 → 1 to module B_1_ and 1 → 0 to module B_2_, as the external input (Fig. 3). These simulations were performed such that the Boolean values ‘true’ represented ‘active’ and ‘false’ represented ‘inactive’ network modules (method is illustrated in Supplementary Fig. S1; the simulation program in Perl language is available in Supplementary Data 4). We defined the state of the human myelomonocytic leukaemia cell transcriptional network under PMA stimulus using 2^8^ = 256-tuple the Boolean value, ‘active’ or ‘inactive’, of the eight modules. The simulation experiments started from all 2^8^ = 256 initial conditions; thus, we produced 2^8^ = 256 cases of perturbation. The simulation experiments were performed on a modular level so that identical expression levels were assigned for the TFs in a module; however, TF-level views of the expression patterns confirmed that the simulation results mimicked actual expression patterns well (assumptions of the model are provided in the Supplementary Note)^15^.

**Figure 4:**
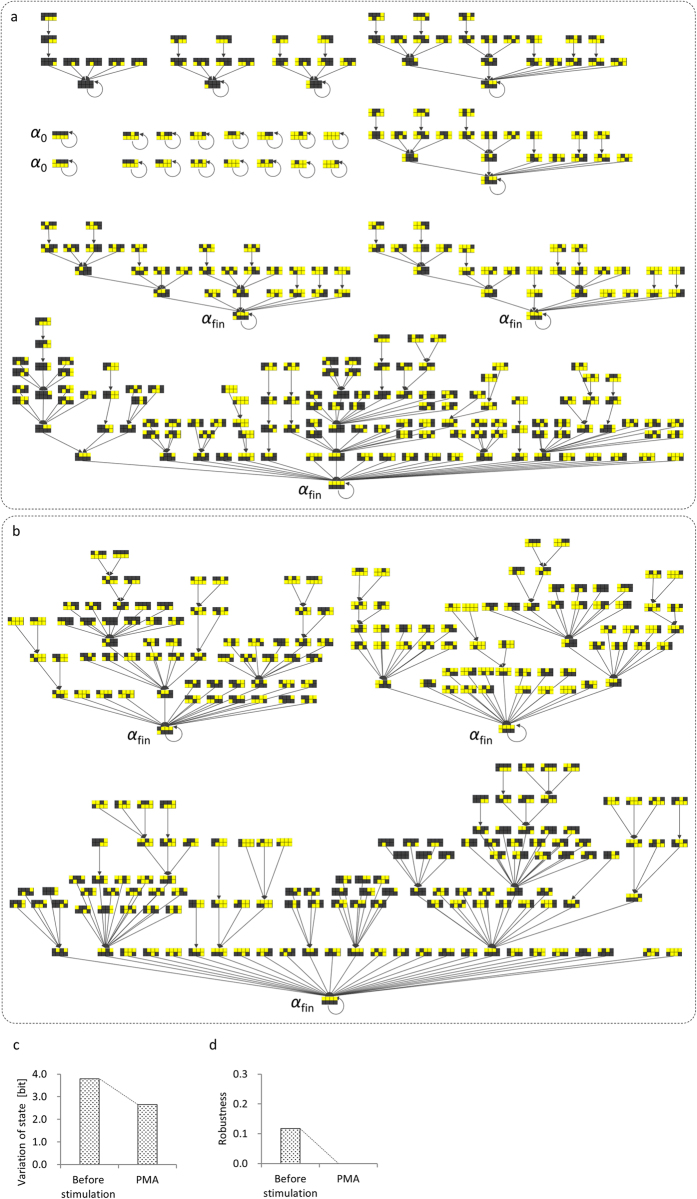
State representation of human myelomonocytic leukaemia cell transcriptional network under phorbol myristate acetate (PMA) stimulus. (**a,b**) State trajectories before and after PMA stimulation^15^, respectively. Step functions (0 → 1 to module B_1_ and 1 → 0 to module B_2_) were supplied to mimic THP-1 cell differentiation under PMA stimulation. States of modular network structure are shown in checkerboards, where upper row, from left to right, indicates states of A_1_, B_1_, C_1_ and D_1_ modules, and lower row indicates states of A_2_, B_2_, C_2_ and D_2_ modules (see Fig. 3). Modules in checkerboards are coded as ‘active’ (yellow) or ‘inactive’ (black). α_fin_ indicates states that mimic the final expression pattern as determined by qRT-PCR; α_0_ indicates states that mimic the initial expression pattern. (**c**) Variation of state measured by bit before and after PMA stimulation. Variation of state was calculated over eight units of time. (**d**) Robustness before and after PMA stimulation.

The simulation results (Fig. 4a,b; state trajectories documented in Supplementary Data 5) showed that states α_fin_ mimicked the final expression pattern determined by quantitative real-time reverse-transcription polymerase chain reaction (qRT-PCR) during human THP-1 cell differentiation under a PMA stimulus^32^, in which the representative profiles of B_1_, C_1_ and D_1_ were upregulated and the representative profiles of B_2_, C_2_ and D_2_ were downregulated compared with their initial expression levels (Fig. 3). States α_0_ (Fig. 4a) mimicked the initial expression patterns in which the representative profiles of B_1_, C_1_ and D_1_ were low, while the representative profiles of B_2_, C_2_ and D_2_ were high compared with their final expression levels (Fig. 3). The states α_0_ with expression patterns that corresponded to the initial expression patterns before PMA stimulation disappeared after PMA stimulation, and an unavoidable convergence to the states α_fin_ is suggested (Fig. 4b). We calculated the variation of state and robustness from the state trajectories. The calculation method was same as that used to calculate the values in the soybean metabolic network (explained in Supplementary Methods; variation of state was calculated over eight units of time for the human THP-1 myelomonocytic leukaemia cell transcriptional network). The variation of state was calculated as 3.80 bit before and 2.65 bit after PMA stimulation (Fig. 4c). The data showed a 14% loss of the theoretical maximum of the variation of state. The robustness decreased from 0.12 to 0 after the PMA stimulus (Fig. 4d; calculation method is explained in Supplementary Methods; robustness was calculated using the states at *t* = 0 and *t* = 8 for the human THP-1 myelomonocytic leukaemia cell transcriptional network). These results suggested that the loss of robustness occurred concurrently with the loss of variation of state in the model under PMA stimulation (Fig. 4c,d).

### Application experiments

**Figure 5:**
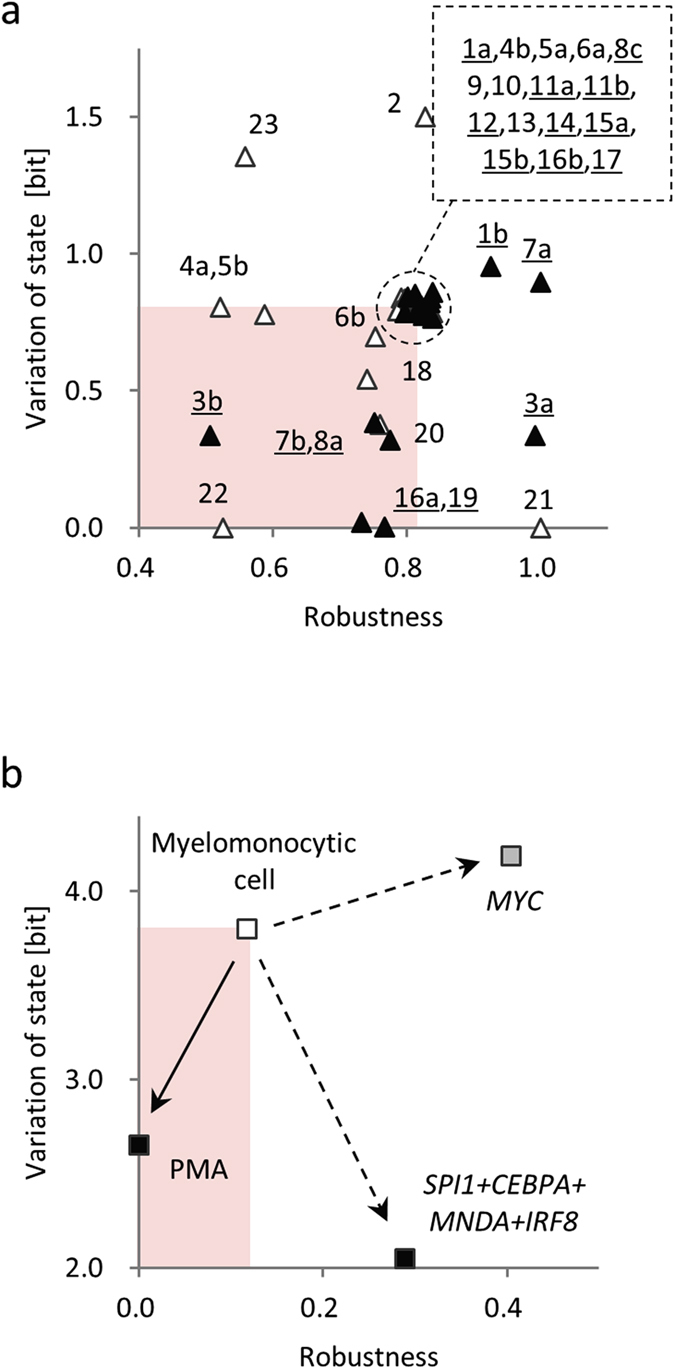
Application simulation experiments. (**a**) Results of simulations for genetic modification. Vertical axis shows variation of state during 4-day simulation period. Alphanumeric symbols attached to triangle indicate reaction number in metabolic data (reaction details are documented in Supplementary Table S1). Filled and open triangles indicate that corresponding enzyme was ‘expressed’ or ‘not expressed’, respectively, in the proteome data^33^,^34^. Underlined alphanumeric symbols indicate that corresponding enzyme was ‘expressed’. A circle-by-dashed line indicates robustness–variation of state pairs that were the same as the value in the control model without over-expression of the enzymes. (**b**) Results of simulations for degree of dedifferentiation. Vertical axis shows variation of state calculated over eight units of time. Open rectangle indicates robustness–variation of state pairs before treatment. Black or grey rectangles indicate robustness–variation of state pairs after treatment that induced THP-1 cell differentiation or dedifferentiation, respectively. Solid arrow indicates robustness–variation of state pair transition by the simulation mimicking extracellular phorbol myristate acetate (PMA) treatment. Dashed arrow indicates robustness–variation of state pair transition by simulation mimicking intracellular enforced expression of key transcription factors. Red shading in both panels indicates where both robustness and variation of state decreased after treatment.

First, we conducted simulation experiments to investigate behavioural changes in the soybean metabolic network resulting from enzyme over-expression (Fig. 1a). One by one, we increased the maximum velocity of each reaction four-fold from the default value in the control model (*V*_*max*_ value documented in Supplementary Table S1), and simulated the state trajectories in each case (documented in Supplementary Data 3; method is explained in Supplementary Methods). Among the 33 cases of enzyme over-expression, four led to a complete loss (=0) of the variation of state, as in the flooded model (Fig. 5a). The simulation experiment results were validated using proteomic data. The enzymes in two of the four cases, glutamate decarboxylase in reaction R19 (Fig. 1a) and alanine aminotransferase in reaction R16 (Fig. 1a), were defined as ‘expressed’ in the proteomic data. Here, we judged a protein as ‘expressed’ if it was present at multiple time points during flooding in the root or cotyledon in the soybean proteome database^33^,^34^. This database contains soybean proteins detected using a gel-free proteomic technique in seedlings from day 2 to day 6 of a flooding treatment. The conditions used for proteomic data collection were the same as those used for metabolic data collection (see Methods). A two-sample test for equality of proportions^35^ showed that the proportion of the cases in which the corresponding protein was observed as ‘expressed’ was statistically significant (*p* = 0.04 in the one-sided test) in the region where variation of state was less than one-half that in the control conditions. Glutamate decarboxylase catalyses the formation of GABA from glutamic acid and is activated by increases in H^+^ or Ca^2+^ ions^36^. Alanine aminotransferase is a hypoxia-induced protein that regulates energy availability in plants under unfavourable environmental conditions^37^. These results suggest that a few enzymes that change plant behaviour can be screened based on the value of the variation of state, and that such enzymes include those related to the abiotic stress response. Thus, the variation of state value is expected to provide genetic targets and is valid as a parameter for use in plant genetic engineering. A binomial test^35^ confirmed the significance of the loss of robustness concurrent with the loss of variation of state. In the simulation cases of enzyme over-expression in which the corresponding protein was observed as ‘expressed’ (filled triangles in Fig. 5a) and the robustness–variation of state pair changed compared with the value in the control model, five out of eight enzyme over-expressions were in the area where both robustness and variation of state were lower than those in the control model. The probability of the event occurring by chance was calculated as *p* = *Combination* (8,5) (1/4)^5^ (1 − 1/4)^3^ = 0.02. This low probability suggested that the loss of robustness concurrent with the loss of variation of state was statistically significant.

Second, we conducted simulation experiments to investigate behavioural changes in the human THP-1 myelomonocytic leukaemia cells resulting from the enforced expression of key TFs in two cases. Case-1 included the *SPI1*, *CEBPA*, *MNDA,* and *IRF*8 TFs that are involved in reconstruction of the monocyte transcriptional regulatory network^38^. Case-2 included *MYC*, a reprogramming Yamanaka factor that may be essential for inducing pluripotent stem cells during dedifferentiation^39^. To conduct the simulations, we added enforced expression to the modules in the transcriptional network (Fig. 3). Case-1 enforced expression of modules A_1_ containing *CEBPA*, A_2_ containing *MNDA*, B_2_ containing *IRF8*, and C_1_ containing *SPI1*. Case-2 enforced expression to module B_2_ containing *MYC*, without supplying the step functions, which models the external PMA input. In the simulations, the enforced expression was mimicked by maintaining the corresponding module in the active state during the simulation period. The results showed that variation of state decreased in the simulations, which mimicked treatments that were thought to induce differentiation of THP-1 cells, from 3.80 to 2.65 (14% decrease of the theoretical maximum) in the PMA stimulation, and from 3.80 to 2.05 (22% decrease of the theoretical maximum) in the enforced expression for reconstruction of the monocyte transcriptional regulatory network (Fig. 5b, state trajectories shown in Supplementary Data 5). The calculation method is explained in Supplementary Methods (the variation of state was calculated over eight units of time and the robustness was calculated using the states at *t* = 0 and *t* = 8 for human THP-1 myelomonocytic leukaemia cell transcriptional network). The variation of state was slightly increased in the simulation mimicking a treatment to induce dedifferentiation of THP-1 cells, from 3.80 to 4.19 (a 5% increase of the theoretical maximum) for enforced expression of *MYC*. These results suggested that the variation of state value can describe the degree of differentiation and may be valid as a parameter in cell biology. When the simulations of extracellular treatment by PMA stimulation (solid arrow in Fig. 5b) and the intracellular treatment by enforced expression of key TFs (dashed arrows in Fig. 5b) were compared, only the former showed a loss of robustness concurrent with the loss of variation of state.

## Discussion

An alternative view of the biological response to the different types of external stimuli in the different organisms is that there is no reference between soybean and human, since they belong to two different kingdoms, plant and animal. In the present study, we applied the approach to both the soybean metabolic profiles and the human myelomonocytic leukaemia cell transcriptional profiles. We described the system states using Boolean representation, and then calculated the variation of state and robustness of the system. We detected a loss of variation of state under external stimuli and a loss of robustness concurrent with the loss of variation of state in both the soybean response to the flooding stimulus and in the human myelomonocytic leukaemia cell response to the PMA stimulus. Thus, this report shows an example of a property shared between two different organisms under two different types of external stimuli.

In the present study, we compared the simulation results of treated conditions (soybean after the flooding stimulus and human myelomonocytic leukaemia cells under the PMA stimulus) with control conditions (soybean before the flooding stimulus and human myelomonocytic leukaemia cell before the PMA stimulus) and then drew conclusions. We consider that these comparisons assure the objectivity of our study.

**Figure 6:**
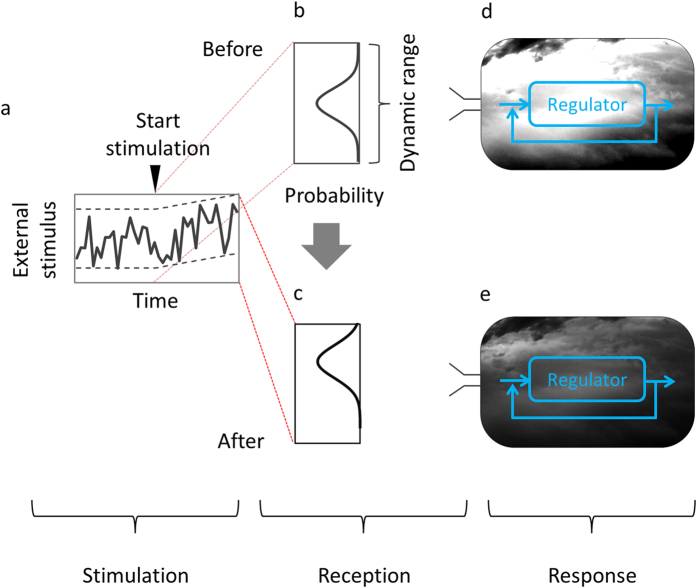
Hypothetical “external stimulus-induced information loss” model of biological systems. (**a**) Temporal profile of external stimulus. (**b**,**c**) Distribution curves of external stimulus. Distribution curve shifts against the dynamic range before (**b**) and after (**c**) external stimulation. (**d**,**e**) Biological system before (**d**) and after (**e**) external stimulation. Biological system has physiological regulation systems. Change in highly varied (**d**) to low-varied (**e**) density in the cloud images represents decrease in the amount of biological system information caused by the external stimulation. Before external stimulation, biological system was filled with rich information (**d**), which was lost after external stimulation (**e**). These cloud images represent the amount of decrease in variation in the state trajectories in the soybean metabolic network under a flooding stimulus, or in the human myelomonocytic leukaemia cell transcriptional network under a phorbol myristate acetate (PMA) stimulus. Number of types of final states that existed before external stimulation (Figs 2a and 4a) decreased in state patterns after external stimulation (Figs 2e and 4b).

In the experimental data that formed the basis of our study, physiological changes were observed or suggested. In soybean, the accumulation of metabolites and increases/decreases in enzyme activity were observed^33^,^34^ or suggested. In human THP-1 myelomonocytic leukaemia cells, changes in gene expression patterns were observed, and alterations in the human THP-1 cell transcriptional network were suggested. Here, we present a hypothetical “external stimulus-induced information loss” model of biological systems that integrates physiological changes and the loss of variation of state observed in our simulations. The hypothetical model is composed of three sub-models: the stimulation, reception, and response sub-models (Fig. 6). In the stimulation sub-model, the temporal profile starts to become biased as result of external stimulation. Bias is a basic property in stochastic processes^40^ and has a net direction and magnitude so that averaging over a large number of observations does not eliminate its effect, unlike a random error^41^. Bias was accounted for in a climate change model in a previously reported environmental study^42^. The reception sub-model connects the stimulation sub-model with the response sub-model. We applied the idea of dynamic range (i.e., a range of stimulus intensities over which a receptor exhibits a response) that has been presented in sensory receptor cell studies^43^,^44^,^45^,^46^. The reception process can be explained as follows: a dynamic range has been optimised through natural selection over generations to maximise the amount of information taken into the biological system in that range; and the amount of information taken into the biological system through the dynamic range decreases according to the bias in the external stimulus. The results of a numerical analysis (illustrated in Supplementary Fig. S2) suggested that the amount of the information taken into the biological system through a dynamic range decreases in accordance with the bias in the external stimulus. In the response sub-model, the information taken into the biological system propagates with physiological changes in the biological system. As a result, the biological system reaches a state. Here, we quantified the biological system-state variation and observed loss of variation in our simulations.

Ito and Sagawa^8^ studied the signal transduction of *Escherichia coli* chemotaxis numerically, and reported a theoretical relationship between information flow in *E. coli* signal transduction and robustness of chemotaxis adaptation against external noise. They described an information thermodynamic relationship in which information flow liberalised the limitation of the robustness of adaptation. Accordingly, the mean square error of the level of adaptation was small because of the transfer entropy in a feedback loop in a microscopic system of signal transduction in *E. coli* chemotaxis^8^. A microscopic system is one in which the magnitude of the thermal fluctuations in system variables is comparable to the magnitude of the system variable. This differs from a macroscopic system in which the magnitude of the thermal fluctuations in system variables is much smaller than the magnitude of the system variable^47^. We extended the relationship between information flow and robustness of adaptation in *E. coli* signal transduction^8^ from the microscopic system to the two macroscopic systems (the soybean metabolic and human myelomonocytic leukaemia cell transcriptional networks) and integrated it with our hypothetical “external stimulus-induced information loss” model of biological systems. Then, we interpreted the loss of robustness (*R*), calculated by equation (2), in the macroscopic systems under the external stimuli applied in our simulations as follows: the loss of all of the information taken into the biological system under the external stimulus caused the loss of variation of state (based on our response sub-model in the hypothetical “external stimulus-induced information loss” model); the loss of all of the information suggested that information flow in the biological system decreased accordingly. This decreased information flow caused degradation of regulation because the reduced robustness of adaptation affected system-states (based on the extension of the relationship) and caused a loss of robustness, *R*. Consequently, we observed the loss of robustness *R* under the external stimuli. The integration of our hypothetical “external stimulus-induced information loss” model of biological systems made consistent the interpretation of the loss of the robustness observed in the soybean metabolic and human myelomonocytic leukaemia cell transcriptional networks, which implied that the hypothetical model was valid.

Environmental response models have been studied in plants. Mooney and Hobbs^48^ reported on the resilience of plants among generations at the level of an individual plant, based on field data. Ponce-Campos *et al.*^49^ reported on the resilience of plants at the ecosystem level. Our study presents an environmental response model of individual soybean plants during their lifetimes (Fig. 6).

Here, we consider the controversial stimulus-triggered acquisition of cell pluripotency (STAP)^50^, based on our results. Obokata *et al.* described STAP cells as lineage-committed lymphocytes and other cells that can be converted to a pluripotent state by altering only the properties of their extracellular environment^51^. Our simulation results, including those obtained under PMA stimulation, suggested that the variation of state decreases under external stimuli regardless of the regulation and stimulus types. Conversely, our simulation results indicated that the treatment thought to induce dedifferentiation of the THP-1 cell by enforced expression of *MYC*, which was suggested to be essential to induce pluripotency of stem cells during dedifferentiation, is a treatment that maintains the variation of state in the biological system (Fig. 5b). These findings suggest that it is unlikely that STAP cells are converted into a pluripotent state only by altering the properties of their extracellular environment.

We generated a hypothetical “external stimulus-induced information loss” model of biological systems by focusing on the transfer of information, which itself is a general entity because it is taken from outside, that propagates in the system and provides the system-states. This report is a step towards understanding biological systems as general systems.

## Methods

### Metabolic data collection from soybean

Soybean seeds (*Glycine max* L. cv. Enrei) were germinated and grown for 6 days. Two-day-old soybean plants were flooded for 4 days and roots including hypocotyls were collected each day during the experiment. The experiments were performed in triplicate. The metabolites were separated and detected using a capillary electrophoresis-mass spectrometry (CE/MS) system (Orbitrap; Thermo Fisher Scientific, San Jose, CA, USA). The concentration of each compound was determined by comparison with a standard curve prepared from known concentrations of a standard compound. The soybean metabolic data collected included temporal profiles of 71 metabolites in plants under flooded and control conditions^23^.

### Transcriptomic data collection from human THP-1 myelomonocytic leukaemia cells

Temporal expression profiles of human THP-1 cells during differentiation were analysed at 0, 1, 2, 4, 6, 12, 24, 48, 72 and 96 hours after starting the PMA treatment, with two biological replicates. The qRT-PCRs were performed using primer pairs generated automatically from a single exon of each target gene. To obtain high-quality transcription data, the reliability and specificity of each primer pair were confirmed in a preliminary experiment. Also, the replicability of the two biological replicates was analysed (see Supplementary Fig. S3). The averaged expression levels (copy numbers) of the two biological replicates were used for further analyses. The expression profiles of 2315 human TFs measured in a THP-1 cell line over a time course of growth, arrest, and differentiation were collected from the Genome Network Platform (http://genomenetwork.nig.ac.jp/index_e.html). After eliminating expression data with suspected measurement errors, we selected 2247 TFs from the 2315 available TFs^15^. Of the 2247 TFs, 1350 (60%) were common to another independently developed dataset of 1962 human TFs^52^.

### Statistical analysis

A Wilcoxon rank sum test^29^ was used to confirm a significant difference in the median of the variation of state between the regions. The test statistic for the Wilcoxon rank sum test was 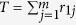, where *r*_1*j*_ is the rank for an observation from group-1 whose sample size is smaller than group-2. In the Wilcoxon rank sum test, *n*_1_ = 12 (the number of data in group-1: *λ* < 0.375) and *n*_2_ = 21 (the number of data in group-2: *λ* ≥ 0.375). A two-sample test for equality of proportions^35^ was used: (i) to identify inter-modular interactions in the transcriptional network modelling; (ii) to confirm a significant difference between the proportions of the cases in which the corresponding protein was considered as ‘expressed’ in the application experiment in which the soybean metabolic network was affected by enzyme over-expression. The test statistic for the two-sample test for equality of proportions was 
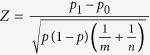
, where 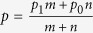, and *m* and *n* are sample sizes. In test (i), *m* ≥ 67^2^ (the number of potential interactions between TFs in the two modules) and *n* = 628^2^ (the number of potential interactions between TFs identified as single TFs^15^). In test (ii), *m* = 9 (the number of cases in the region where the variation of state was less than one-half that in the control conditions) and *n* = 8 (the number of cases in the region where the variation of state was equal to or greater than one-half that in the control conditions). A binomial test^35^ was used to confirm the loss of robustness concurrent with the loss of variation of state in the application experiment in which the soybean metabolic network was affected by enzyme over-expression. In the binomial test, the sample size was 8 (the number of cases in which the corresponding protein was observed as ‘expressed’ and the robustness–variation of the state pair differed from the value in the control model). In the present study, *p* < 0.05 was considered significant.

## Additional Information

**How to cite this article**: Sakata, K. *et al.* Loss of variation of state detected in soybean metabolic and human myelomonocytic leukaemia cell transcriptional networks under external stimuli. *Sci. Rep.* **6**, 35946; doi: 10.1038/srep35946 (2016).

## Supplementary Material

Supplementary Information

Supplementary Information

Supplementary Information

Supplementary Data 1

Supplementary Note 1

Supplementary Data 2

## References

[b1] Schrödinger E. What is life? The physical aspect of the living cell (Cambridge University Press, 1944).

[b2] Simon H. A. The architecture of complexity. Proc. Am. Phil. Soc. 106, 467–482 (1962).

[b3] Von Neumann J. Re-evaluation of the problems of complicated automata-problems of hierarchy and evolution in Theory of self-reproducing automata (ed. Burks A. W.) 74–87 (University of Illinois Press, 1966).

[b4] Weng G., Bhalla U. S. & Iyengar R. Complexity in biological signaling systems. Science 284, 92–96 (1999). 10102825 10.1126/science.284.5411.92 PMC3773983

[b5] Cohen I. R. & Harel D. Explaining a complex living system: dynamics, multi-scaling and emergence. J. R. Soc. Interface 4, 175–182 (2007). 17251153 10.1098/rsif.2006.0173 PMC2359859

[b6] Nagano A. J. *et al.* Deciphering and prediction of transcriptome dynamics under fluctuating field conditions. Cell 151, 1358–1369 (2012). 23217716 10.1016/j.cell.2012.10.048

[b7] Uda S. *et al.* Robustness and compensation of information transmission of signaling pathways. Science 341, 558–561 (2013). 23908238 10.1126/science.1234511

[b8] Ito S. & Sagawa T. Maxwell’s demon in biochemical signal transduction with feedback loop. Nat. Commun. 6, 7498 (2015). 26099556 10.1038/ncomms8498 PMC4557369

[b9] Emmeche C. Aspects of complexity in life and science. Philosophica 59, 41–68 (1997).

[b10] Crofts A. R. Life, information, entropy, and time: vehicles for semantic inheritance. Complexity 13, 14–50 (2007). 18978960 10.1002/cplx.20180 PMC2577055

[b11] Hirayama T. & Shinozaki K. Research on plant abiotic stress responses in the post-genome era: past, present and future. Plant J. 61, 1041–1052 (2010). 20409277 10.1111/j.1365-313X.2010.04124.x

[b12] Komatsu S., Shirasaka N. & Sakata K. ‘Omics’ techniques for identifying flooding-response mechanisms in soybean. J. Proteomics 93, 169–178 (2013). 23313220 10.1016/j.jprot.2012.12.016

[b13] Komatsu S., Hiraga S. & Yanagawa Y. Proteomics techniques for the development of flood tolerant crops. J. Proteome Res. 11, 68–78 (2012). 22029422 10.1021/pr2008863

[b14] Kauffman S. Differentiation: the dynamical behaviors of genetic regulatory networks in The origins of order: self-organization and selection in evolution, Ch. 12, 441–522 (Oxford University Press, 1993).

[b15] Sakata K. *et al.* System-wide analysis of the transcriptional network of human myelomonocytic leukemia cells predicts attractor structure and phorbol-ester-induced differentiation and dedifferentiation transitions. Sci. Rep. 5, 8283 (2015). 25655563 10.1038/srep08283 PMC4319166

[b16] Tsuchiya S. *et al.* Induction of maturation in cultured human monocytic leukemia cells by a phorbol diester. Cancer Res. 42, 1530–1536 (1982). 6949641

[b17] Abrink M., Gobl A. E., Huang R., Nilsson K. & Hellman L. Human cell lines U-937, THP-1 and Mono Mac 6 represent relatively immature cells of the monocyte macrophage cell lineage. Leukemia 8, 1579–1584 (1994). 8090034

[b18] Ashburner M. *et al.* Gene ontology: tool for the unification of biology. Nat. Genet. 25, 25–29 (2000). 10802651 10.1038/75556 PMC3037419

[b19] Shannon C. E. A mathematical theory of communication. *Bell Syst*. Technical J. 27, 379–423 (1948).

[b20] Smit B., Burton I., Klein R. J. T. & Street R. The science of adaptation: a framework for assessment. Mitigation Adapt. Strateg. Glob. Chang. 4, 199–213 (1999).

[b21] Assessment of adaptation practices, options, constraints and capacity in Climate change 2007: impacts, adaptation and vulnerability (eds Parry, M. L., Canziani, O. F., Palutikof, J. P., van der Linden, P. J. & Hanson, C. E.) Ch. 17, 717–744 (Cambridge University Press, 2007).

[b22] Givant S. & Halmos P. Boolean algebras in Introduction to Boolean algebras (ed. Axler G. & Ribet K. A.) 8–13 (Springer, 2009).

[b23] Nakamura T. *et al.* Evaluation of metabolite alteration under flooding stress in soybeans. Jpn. Agric. Res. Q. 46, 237–248 (2012).

[b24] Bouché N. & Fromm H. GABA in plants: just a metabolite? Trends Plant Sci. 9, 110–115 (2004). 15003233 10.1016/j.tplants.2004.01.006

[b25] Fait A., Fromm H., Walter D., Galili G. & Fernie A. R. Highway or byway: the metabolic role of the GABA shunt in plants. Trends Plant Sci. 13, 14–19 (2008). 18155636 10.1016/j.tplants.2007.10.005

[b26] Kinnersleya A. M. & Turano F. J. Gamma aminobutyric acid (GABA) and plant responses to stress. Crit. Rev. Plant Sci. 19, 479–509 (2000).

[b27] Schomburg I. *et al.* BRENDA, the enzyme database: updates and major new developments. Nucl. Acids Res. 32, D431–D433 (2004). 14681450 10.1093/nar/gkh081 PMC308815

[b28] Sekiguchi T. & Okamoto M. Winbest-kit: windows-based biochemical reaction simulator for metabolic pathways. J. Bioinform Comput. Biol. 4, 621–638 (2006). 16960966 10.1142/s0219720006002132

[b29] Whitley E. & Ball J. Statistics review 6: nonparametric methods. Crit Care. 6, 509–513 (2002). 12493072 10.1186/cc1820 PMC153434

[b30] Tritchler D., Parkhomenko E. & Beyene J. Filtering genes for cluster and network analysis. BMC Bioinformatics 10, 193 (2009). 19549335 10.1186/1471-2105-10-193 PMC2708160

[b31] Gupta A., Maranas C. D. & Albert R. Elucidation of directionality for co-expressed genes: predicting intra-operon termination sites. Bioinformatics 22, 209–214 (2006). 16287937 10.1093/bioinformatics/bti780

[b32] The FANTOM Consortium and the Riken Omics Science Center. The transcriptional network that controls growth arrest and differentiation in a human myeloid leukemia cell line. Nat. Genet. 41, 553–562 (2009). 19377474 10.1038/ng.375 PMC6711855

[b33] Sakata K. *et al.* Soybean proteome database: a data resource for plant differential omics. J. Proteome Res. 8, 3539–3548 (2009). 19489578 10.1021/pr900229k

[b34] Ohyanagi H., Sakata K. & Komatsu S. Soybean Proteome Database 2012: update on the comprehensive data repository for soybean proteomics. Front. Plant Sci. 3, 110 (2012). 22661982 10.3389/fpls.2012.00110 PMC3362740

[b35] Moore D. S. From exploration to inference and Inference about variables in The basic practice of statistics (ed. Bleyer C.) 198–557 (W. H. Freeman & Company, 2010).

[b36] Ramputh A. I. & Bown A. W. Rapid γ-aminobutyric acid synthesis and the inhibition of the growth and development of oblique-banded leaf-roller larvae. Plant Physiol. 111, 1349–1352 (1996). 12226367 10.1104/pp.111.4.1349 PMC161023

[b37] Kendziorek M., Paszkowski A. & Zagdanska B. Differential regulation of alanine aminotransferase homologues by abiotic stresses in wheat (*Triticum aestivum* L.) seedlings. Plant Cell Rep. 31, 1105–1117 (2012). 22327955 10.1007/s00299-012-1231-2 PMC3351597

[b38] Suzuki T. *et al.* Reconstruction of monocyte transcriptional regulatory network accompanies monocytic functions in human fibroblasts. PLoS One 7, e33474 (2012). 22428058 10.1371/journal.pone.0033474 PMC3302774

[b39] Takahashi K. *et al.* Induction of pluripotent stem cells from adult human fibroblasts by defined factors. Cell 131, 861–872 (2007). 18035408 10.1016/j.cell.2007.11.019

[b40] Shlesinger M. F. & Landman U. Solutions of physical stochastic processes via mapping onto ideal and defective random walk lattices in Applied Stochastic Processes (ed. Adomian G.) 151–246 (Academic Press, 1980).

[b41] Department of Statistics Online Programs. Lesson 4: Bias and Random Error in *STAT 509: Design and analysis of clinical trials*, https://onlinecourses.science.psu.edu/stat509/node/26 (2016).

[b42] Smit B. & Wandel J. Adaptation, adaptive capacity and vulnerability. Glob. Environ. Chang. 16, 282–292 (2006).

[b43] Crawford A. C., Evans M. G. & Fettiplace R. Activation and adaptation on transducer currents in turtle hair cells. J Physiol. 419, 405–434 (1989). 2621635 10.1113/jphysiol.1989.sp017878 PMC1190013

[b44] Forti S., Menini A., Rispoli G. & Torre V. Kinetics of phototransduction in retinal rods of the newt *Triturus cristutus*. J Physiol. 419, 265–295 (1989). 2621632 10.1113/jphysiol.1989.sp017873 PMC1190008

[b45] Menini A., Picco C. & Firestein S. Quantal-like current fluctuations induced by odorants in olfactory receptor cells. Nature 373, 435–437 (1995). 7830795 10.1038/373435a0

[b46] Torre V., Ashmore J. F., Lamb T. D. & Menini A. Transduction and adaptation in sensory receptor cells. J. Neurosci. 15, 7757–7768 (1995). 8613717 10.1523/JNEUROSCI.15-12-07757.1995 PMC6577959

[b47] Lavenda B. H. From one to infinity in Statistical physics: a probabilistic approach. 111–166 (Wiley, 1991).

[b48] Mooney H. A. & Hobbs R. J. Resilience at the individual plant level in Resilience in Mediterranean-type ecosystems, Vol. 16 (eds Dell B., Hopkins A. J. M. & Lamont B. B.) 65–82 (Springer: Netherlands, , 1986).

[b49] Ponce-Campos G. E. *et al.* Ecosystem resilience despite large-scale altered hydroclimatic conditions, Nature 494, 349–352 (2013). 23334410 10.1038/nature11836

[b50] *The rise and fall of STAP*. (2015) Available at: www.nature.com/news/stap-1.15332. (Accessed: 2nd May 2016).

[b51] Baumann K. Stem cells: reprogramming with low pH. Nat. Rev. Mol. Cell Biol. 15, 149 (2014). 24518367 10.1038/nrm3754

[b52] Messina D. M., Glassock J., Gish W. & Lovett M. An ORFeome-based analysis of human transcription factor genes and the construction of a microarray to interrogate their expression. Genome Res. 14, 2041–2047 (2004). 15489324 10.1101/gr.2584104 PMC528918

