# Supplementary material for: Loss of variation of state detected in soybean metabolic and human myelomonocytic leukaemia cell transcriptional networks under external stimuli
Source: Sci Rep. 2016 Oct 24;6:35946. doi: 10.1038/srep35946 (PMC5075873; doi:10.1038/srep35946)
Supplement: Supplementary Information [file srep35946-s1.pdf]

Supplementary Information

Loss of variation of state detected in soybean metabolic and human myelomonocytic leukaemia cell transcriptional networks under external stimuli

Katsumi Sakata[1,2,*], Toshiyuki Saito[2], Hajime Ohyanagi[2,3], Jun Okumura[1], Kentaro Ishige[1], Harukazu Suzuki[4], Takuji Nakamura[5], and Setsuko Komatsu[6]

[1] Maebashi Institute of Technology, Maebashi 371-0816, Japan
[2] National Institute of Radiological Sciences, Chiba 263-8555, Japan
[3] King Abdullah University of Science and Technology, Thuwal 23955-6900, Kingdom of Saudi Arabia
[4] RIKEN Centre for Life Science Technologies, Yokohama 230-0045, Japan
[5] National Agriculture and Food Research Organisation (NARO) Hokkaido Agricultural Research Centre, Sapporo 062-8555, Japan
[6] NARO Institute of Crop Science, Tsukuba 305-8518, Japan

[*] Corresponding author: ksakata@maebashi-it.ac.jp

Table S1. Reactions in differential equation model of soybean metabolic system used in this study

Reaction	Substrate	Product	Kinetics[1]	K_m [μmol g DW^{-1}]	V_{max}-control [μmol g DW^{-1} h^{-1}]	V_{max}-flooded [μmol g DW^{-1} h^{-1}]
R1a	Citrate	Isocitrate	Michaelis–Menten	0.12	0.958	0.708
R1b	Isocitrate	Citrate	Michaelis–Menten	0.58	0.625	0.313
R2	Isocitrate	2OG	Michaelis–Menten	0.12	0.542	0.375
R3a	2OG	Succinyl-CoA	Michaelis–Menten	0.25	1.208	0.333
R3b	Succinyl-CoA	2OG	Michaelis–Menten	0.25	0.125	0.625
R4a	Succinyl-CoA	Succinate	Michaelis–Menten	0.086	0.625	0.708
R4b	Succinate	Succinyl-CoA	Michaelis–Menten	0.49	0.313	0.417
R5a	Succinate	Fumarate	Michaelis–Menten	0.23	0.75	0.75
R5b	Fumarate	Succinate	Michaelis–Menten	0.455	0.208	0.583
R6a	Fumarate	Malate	Michaelis–Menten	0.031	0.708	0.875
R6b	Malate	Fumarate	Michaelis–Menten	0.45	0.208	0.375
R7a	Malate	OAA	Michaelis–Menten	0.19	1.042	0.813
R7b	OAA	Malate	Michaelis–Menten	0.0121	0.208	0.208
R8a	OAA	Citrate	Michaelis–Menten	0.016	0.313	0.49
R8b	Citrate	Acetyl-CoA, OAA	Michaelis–Menten	0.1	0.042	0.208
R8c	Acetyl-CoA	Citrate	Michaelis–Menten	0.031	0.292	1.042
R9	Asp	Fumarate	Michaelis–Menten	2.6	0.125	0.938
R10	Asp	Ala	Michaelis–Menten	1.3	0.021	0.729
R11a	Alcohol	Acetaldehyde	Michaelis–Menten	3.5	0.208	0.208
R11b	Acetaldehyde	Alcohol	Michaelis–Menten	1.45	0.208	0.208
R12	Pyr	Acetaldehyde	Michaelis–Menten	0.042	0.063	0.063
R13	Pyr	Acetyl-CoA	Michaelis–Menten	0.09	0.021	0
R14	PEP	Pyr	Michaelis–Menten	0.052	0.208	0.208
R15a	Pyr	Lac, LDHi	Competitive inhibition	0.69	0.021	7.292
R15b	Lac	Pyr	Michaelis–Menten	19.5	0.042	0.833
R15c	xi	LDHi	Mass action[2]	Not applicable	Not applicable	Not applicable
R16a	Ala, 2OG	Pyr, Glu	Ping-pong	2.8, 0.28[3]	0.417	0.417
R16b	Pyr, H^+	Ala	Ordered	0.09, 2.8[3]	0.208	0.625
R16c	Ala, NAD	Pyr, H^+	Ordered	0.37, 0.36[3]	0.417	0.313
R17	Glu, OAA	2OG, Asp	Ordered	0.023, 7.5[3]	0.417	0.458
R18	OAA	PEP	Michaelis–Menten	0.016	0.021	0.042
R19	Glu, H^+	GABA	Ordered	22, 22[3]	4.625	4.792
R20	SSA	2OG	Michaelis–Menten	0.33	0.333	0.417
R21	GABA	SSA	Michaelis–Menten	0.18	0.5	0.438
R22	Gln, 2OG	Glu	Ordered	0.24, 0.022[3]	0.25	0.396
R23	SSA	Succinate	Michaelis–Menten	1.06	0.052	0.208
R24	GTP, OAA	GDP, EP	Ordered	0.065, 0.016[3]	0.375	0.208

[1] We assigned kinetic functions based on data provided on the web page for each enzyme in BRENDA database[27] and/or Purich (2010)[53] ; [2] k=0.417 [h^{-1}]; [3] *KmA*, *KmB*

Table S2. Reactants in differential equation model of soybean metabolic system used in this study

Reactant	Type	Initial value[1] [μmol gDW^{-1}]
2OG	Variable	0.295
Acetaldehyde	Variable	1
Acetyl-CoA	Variable	0.011
Ala	Variable	8.85
Alcohol	Variable	1
Asp	Pool	4.339
Citrate	Variable	12.1
EP	Variable	1
Fumarate	Variable	2.7
GABA	Variable	5.34
GDP	Variable	0.034
Gln	Pool	1.1
Glu	Variable	5.6
GTP	Pool	1.34
H^+	Pool	20
Isocitrate	Variable	2.84
Lactate	Variable	0.676
LDHi	Variable	0
Malate	Variable	44
NAD	Pool	0.0011
OAA	Variable	1.8
PEP	Pool	0.157
Pyr	Pool	0.94
SSA	Variable	5
Succinate	Variable	1.34
Succinyl-CoA	Variable	1
xi	Pool	7

[1] We set initial values based on experimental data from 2-day-old soybean plants.

Table S3. Kinetic equations used in differential equation model of soybean metabolic system

Competitive inhibition	$v = \frac{V_{\max}[S]}{K_m^{app} + [S]}, K_m^{app} = K_m\left(1 + \frac{[I]}{K_i}\right)$
Mass action	$v = k[S]$
Michaelis–Menten	$v = \frac{V_{\max}[S]}{K_m + [S]}$
Ordered	$v = \frac{V_{\max}[A][B]}{K_A K_B + K_B[A] + [A][B]}$
Ping-pong	$v = \frac{V_{\max}[A][B]}{K_B[A] + K_A[B] + [A][B]}$

a

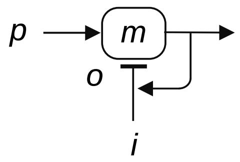

b

Module	Inhibitory input	Operator
0	0	0
0	1	1
1	0	0
1	1	0

Promotive input	Operator	Module
0	0	0
0	1	0
1	0	1
1	1	0

Figure S1. State trajectory simulation of modular human THP-1 cells transcriptional network based on Boolean functions (modified from Kauffman[14]). (**a**) Schematic of Boolean functions used in simulation experiments. *p* is promoting input, *i* is inhibitory input, *o* is operator, and *m* is transcription factor module. Directed edges from *p* and *i* are indicated by red arrows and blue T-bars, respectively, in Fig. 3. Directed edge from *i* blocks activation of the module unless it is itself inhibited by activation of the module. (**b**) Effect of "NOT IF" Boolean function. Table (left) shows "NOT IF" Boolean function describing regulation of an operator by a modular state and inhibitory input. For an operator (*o*), 1 = enabled and 0 = disabled. For a module (*m*), 1 = active and 0 = inactive. Table (right) shows "NOT IF" Boolean function describing activation of a module. Activation requires that promotive input (*p*) is active (1), and operator is disabled (0). In Fig. 3, multiple T-bars to a module are considered as at least one input (1) that guarantees inhibition is enabled (1). In upper modules (A_1, B_1, C_1, and D_1) in Fig. 3, multiple arrows to a module are considered as at least one input (1) that guarantees promotion is enabled (1). In lower modules (A_2, B_2, C_2, and D_2) in Fig. 3, all inputs (1) are required to guarantee that promotion is enabled (1)[15].

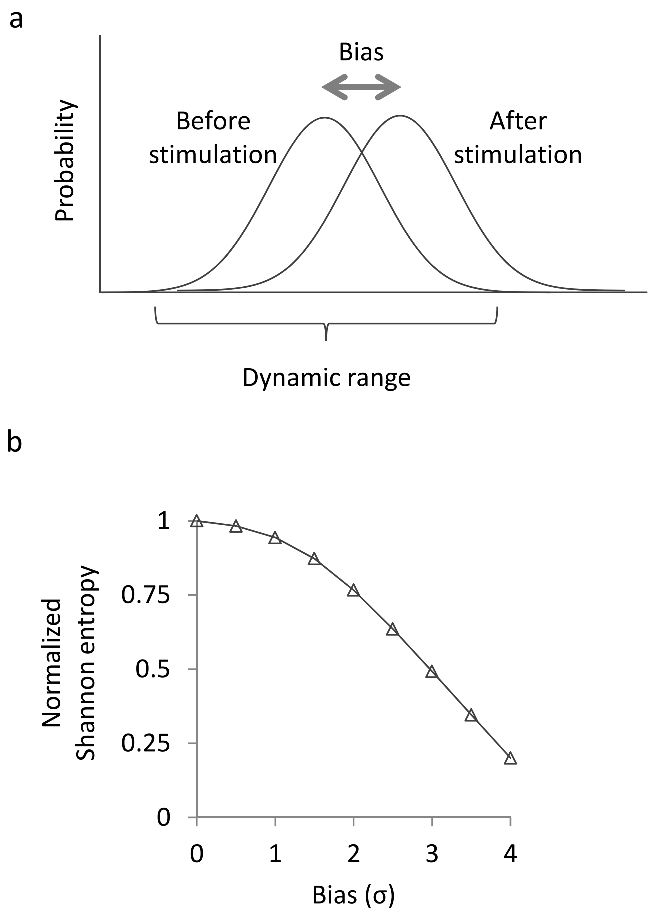

Figure S2. Information loss induced by external stimulus bias in hypothetical "external stimulus-induced information loss" model of biological systems. (**a**) External stimulus distribution before and after external stimulation against a dynamic range. (**b**) Loss of normalised Shannon entropy, which represents the amount of information taken into the system through the dynamic range, induced by the external stimulus bias shown in panel (**a**). In this simulation, we assumed that the external stimulus had a normal distribution whose mean corresponded to the centre of the dynamic range and that the dynamic range size was six times (standard deviation of normal distribution).

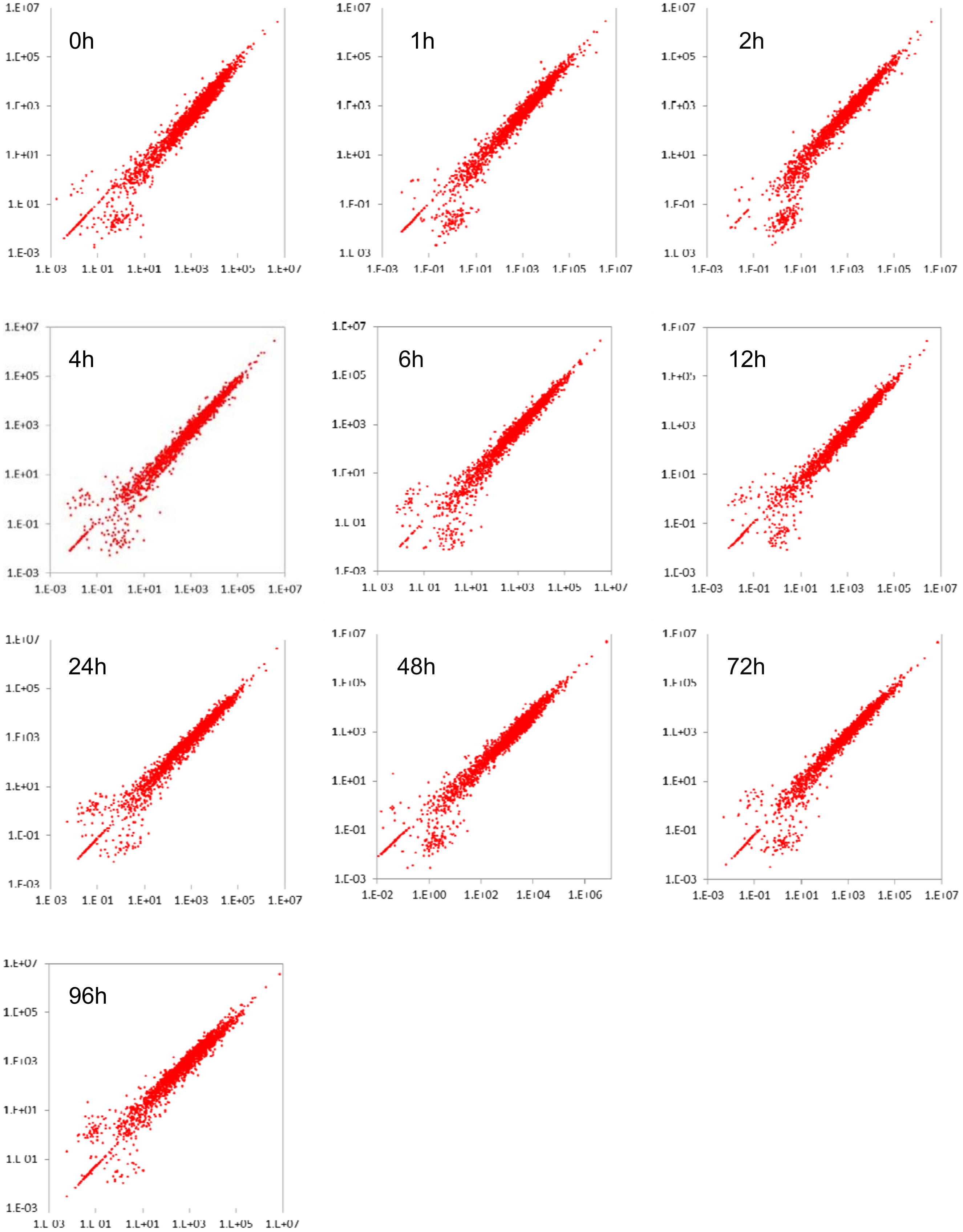
0h
1h
2h
4h
6h
12h
24h
48h
72h
96h

Figure S3. Scatter plots between expression levels at each time point determined by qRT-PCR of 2247 TFs in two biological replicates. Horizontal and vertical axes show expression level in first and second replicate, respectively. Spearman correlations between TF expression levels in the two replicates were 0.980, 0.987, 0.987, 0.989, 0.989, 0.988, 0.988, 0.984, 0.990 and 0.981, for 0, 1, 2, 4, 6, 12, 24, 48, 72, and 96 h time points, respectively. These Spearman correlation values were higher than those (0.65-0.70) calculated for expression data from human myelomonocytic leukaemia cells using cap analysis of gene expression[32], indicating high replicability of expression data used in this study.

Supplementary Note

Growth suppression in soybean seedling after flooding

Physiological changes were evaluated in the early stages of flooding stress[54]. For these analyses, soybean seeds were germinated on sand for 2 days and then subjected to flooding for 4 days. The length of the lateral and adventitious roots and the overall growth of the plants was suppressed after 1 day of flooding stress. The root growth suppression continued over the 4 days. The fresh weight of the hypocotyl and total roots was 50% lower in flooding-stressed plants than in control plants. The main root was 25% shorter in flooding-stressed plants than in control plants[54].

Maximum entropy in multi-state system

The maximum value for $H(X) = -\sum_{i=1}^{n} P(x_i)\log P(x_i)$ under the constraint $\sum_{i=1}^{n} P(x_i) = 1$ was given by the Lagrange multipliers method[55]. The Lagrange function is as follows:

$L = -\sum_{i=1}^{n} P(x_i)\log P(x_i) + \lambda\left(\sum_{i=1}^{n} P(x_i) - 1\right)$. We solved $\frac{\partial L}{\partial P(x_i)} = -\log P(x_i) - 1 + \lambda = 0$.

This equation means that $P(x_1) = P(x_2) = \cdots = P(x_n)$ for $i = 1, 2, \cdots, n$. Using the constraint $\sum_{i=1}^{n} P(x_i) = 1$, we obtained the solution $P(x_i) = 1/n \quad (i = 1, 2, \cdots, n)$, and maximum $H(X) = -\log(1/n)$.

Assumptions of transcriptional regulation model

The transcriptional regulation model (Fig. S1) is based on the following assumptions: the state ('active' or 'inactive') of a gene module induces the state ('active' or 'inactive') of another gene module; Boolean functions represent this relationship between gene modules. Huang and Ingber showed that simulations based on a Boolean network model of individual genes well-mimicked expression patterns in human endothelial cell growth and quiescence[56]. Huang *et al.* suggested the existence of gene modules in mouse hematopoietic stem cells in which two groups of genes that were expressed in the undifferentiated cells and whose expression changed in opposite directions as cells differentiated into erythroid and myelomonocytic lineages (i.e., genes are down-regulated in one lineage and up-regulated in the other[57]). Shu *et al.* revealed a regulatory network composed of individual genes, gene modules, and activation or inhibition interactions between network-nodes (genes and gene modules) from experimental data of induction of pluripotency in mouse somatic cells[58]. These previous studies suggest the validity of our regulation model based on transcriptional modules (Fig. S1a) and Boolean functions (Fig. S1b). Furthermore, in our previous study, we compared simulation results produced using a modular regulation model so that identical expression levels were assigned for TFs in a module, with actual expression levels of individual TFs. We

visualised the transcriptional network composed of ~1,600 TFs and indicated the expression level of each TF in the network by colour densities, and confirmed visually that simulation results well-mimicked actual expression patterns[15].

Supplementary Methods

Calculation of variation of state *H*(*X*) and robustness *R*(*X*) from state trajectories

Here, we describe the methods based on an example. The graphs below show an example of the temporal profiles of the simulated amounts of 2OG, Glu, GABA, and succinate in soybean. In the simulation, the amount of metabolites was updated every 0.04 day (=0.96 hour) based on the differential equation model (see Supplementary Table S1). A plus symbol indicates the sampling point of the simulation results. At each sampling time (0, 1, 2, 3, and 4 days), the amount of each metabolite was translated into Boolean representation as ‘true’, representing ‘accumulated’ (more than double the initial amount of metabolite shown in Supplementary Table S2) or ‘false’, representing ‘not accumulated’ (no increase or an increase to less than double the initial amount). Red shading shows the area where the amount of the metabolite is more than double the initial amount of metabolite shown in Supplementary Table S2. The number 0 or 1 indicates the Boolean value translated from the simulated amount of each metabolite at the corresponding sampling time. In the following diagrams, the initial value of 2OG was set to double the initial value, and other metabolites were set to the same as the initial value in Supplementary Table S2.

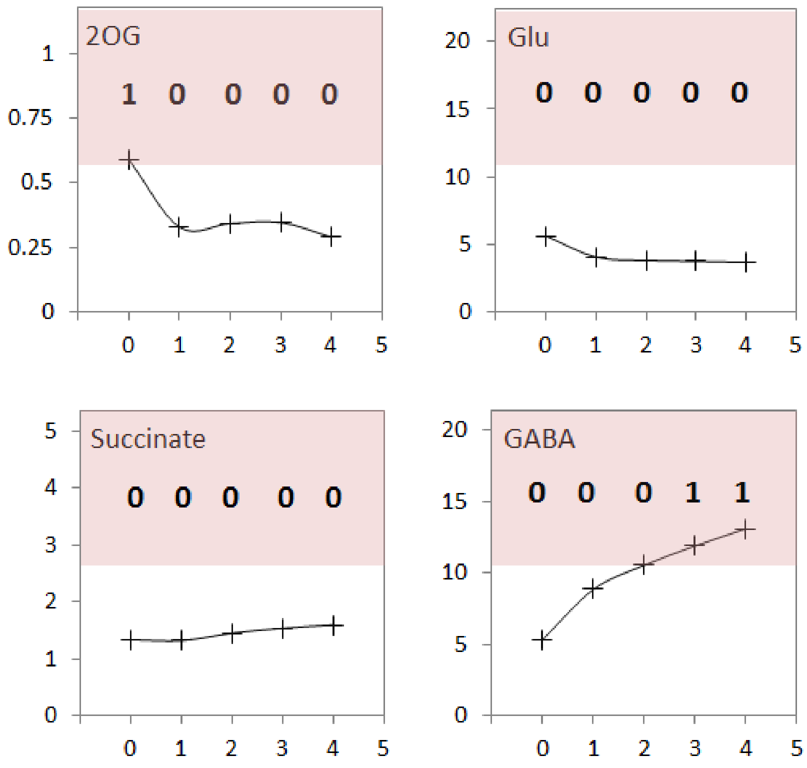

The following tables show the state trajectories of four metabolites; 2OG, Glu, GABA and Succinate in a Boolean representation. Trajectories start from 16 ($=2^4$) initial states ($t=0$, grey in the tables). The table in the red box corresponds to the state trajectory of the example explained

above. The state (0 0 0 0) indicated by a black rectangle represents the state without perturbation. The initial (t=0) and final (t=4) states were plotted in Fig. 2a–e in main text.

t	2OG	Glu	GABA	Succinate
0	0	0	0	0
1	0	0	0	0
2	0	0	0	0
3	0	0	1	0
4	0	0	1	0

t	2OG	Glu	GABA	Succinate
0	0	0	0	1
1	0	0	0	0
2	0	0	0	0
3	0	0	1	0
4	0	0	1	0

t	2OG	Glu	GABA	Succinate
0	0	0	1	0
1	0	0	1	0
2	0	0	1	0
3	0	0	1	0
4	0	0	1	0

t	2OG	Glu	GABA	Succinate
0	0	0	1	1
1	0	0	1	0
2	0	0	1	0
3	0	0	1	0
4	0	0	1	0

t	2OG	Glu	GABA	Succinate
0	0	1	0	0
1	0	0	1	0
2	0	0	1	0
3	0	0	1	0
4	0	0	1	0

t	2OG	Glu	GABA	Succinate
0	0	1	0	1
1	0	0	1	0
2	0	0	1	0
3	0	0	1	0
4	0	0	1	0

t	2OG	Glu	GABA	Succinate
0	0	1	1	0
1	0	0	1	0
2	0	0	1	0
3	0	0	1	0
4	0	0	1	0

t	2OG	Glu	GABA	Succinate
0	0	1	1	1
1	0	0	1	0
2	0	0	1	0
3	0	0	1	0
4	0	0	1	0

t	2OG	Glu	GABA	Succinate
0	1	0	0	0
1	0	0	0	0
2	0	0	0	0
3	0	0	1	0
4	0	0	1	0

t	2OG	Glu	GABA	Succinate
0	1	0	0	1
1	0	0	0	0
2	0	0	0	0
3	0	0	1	0
4	0	0	1	0

t	2OG	Glu	GABA	Succinate
0	1	0	1	0
1	0	0	1	0
2	0	0	1	0
3	0	0	1	0
4	0	0	1	0

t	2OG	Glu	GABA	Succinate
0	1	0	1	1
1	0	0	1	0
2	0	0	1	0
3	0	0	1	0
4	0	0	1	0

t	2OG	Glu	GABA	Succinate
0	1	1	0	0
1	0	0	1	0
2	0	0	1	0
3	0	0	1	0
4	0	0	1	0

t	2OG	Glu	GABA	Succinate
0	1	1	0	1
1	0	0	1	0
2	0	0	1	0
3	0	0	1	0
4	0	0	1	0

t	2OG	Glu	GABA	Succinate
0	1	1	1	0
1	0	0	1	0
2	0	0	1	0
3	0	0	1	0
4	0	0	1	0

t	2OG	Glu	GABA	Succinate
0	1	1	1	1
1	0	0	1	0
2	0	0	1	0
3	0	0	1	0
4	0	0	1	0

Variation of state $H(X)$ was calculated using state trajectories on four units of time (t=1, 2, 3, and 4): P (0 0 0 0)=8/64=0.125 (blue), P (0 0 1 0)=56/64=0.875 (orange) and $H(X)=-0.125\times log_2 0.125-0.875\times log_2 0.875=0.54$ [bit].

Robustness $R(X)$ was calculated using unperturbed state (t=0, black rectangle in first table) and sixteen final states (t=4) in the trajectories:

$$P(x \mid x_{conv}^{\text{2OG}} = x_{bef}^{\text{2OG}}) = 16/16 = 1,\ P(x \mid x_{conv}^{\text{Glu}} = x_{bef}^{\text{Glu}}) = 16/16 = 1,\ P(x \mid x_{conv}^{\text{GABA}} = x_{bef}^{\text{GABA}}) = 0/16 = 0$$

and $P(x \mid x_{conv}^{\text{Succinate}} = x_{bef}^{\text{Succinate}}) = 16/16 = 1$. $R(X) = \left(1+1+0+1\right)/4 = 0.75$.

Additional References for Supplementary Information (reference number continued from main text)

53. Purich, D. L. *Enzyme kinetics: catalysis and control: a reference of theory and best-practice methods* (Elsevier, 2010).
54. Komatsu, S. *et al.* A comprehensive analysis of the soybean genes and proteins expressed under flooding stress using transcriptome and proteome techniques. *J. Proteome Res.* **8**, 4766-4778 (2009).
55. Arfken, G. B. & Weber, H. J. Lagrange Multipliers in *Mathematical methods for physicists, 3rd ed.* (ed. Singer, T.) 945-950 (Academic Press, 1985).
56. Huang, S. & Ingber, D. E. Shape-dependent control of cell growth, differentiation, and apoptosis: switching between attractors in cell regulatory networks. *Exp Cell Res.* **261**, 91-103 (2000).
57. Huang, S., Guo, Y.-P., May, G. & Enver, T. Bifurcation dynamics in lineage-commitment in bipotent progenitor cells. *Dev Biol.* **305**, 695-713 (2007).
58. Shu, J. *et al.* Induction of pluripotency in mouse somatic cells with lineage specifiers. *Cell* **153**, 963-975 (2013).
